# Possible Impacts of the Invasive Plant *Rubus niveus* on the Native Vegetation of the Scalesia Forest in the Galapagos Islands

**DOI:** 10.1371/journal.pone.0048106

**Published:** 2012-10-31

**Authors:** Jorge Luis Rentería, Mark R. Gardener, F. Dane Panetta, Rachel Atkinson, Mick J. Crawley

**Affiliations:** 1 Department of Biology, Imperial College London, Silwood Park Campus, Ascot, Berkshire, United Kingdom; 2 Charles Darwin Foundation, Puerto Ayora, Galapagos Islands, Ecuador; 3 School of Plant Biology, The University of Western Australia, Crawley, Western Australia, Australia; 4 Invasive Plant Science, Biosecurity Queensland, Department of Employment, Economic Development and Innovation, Ecosciences Precinct, Brisbane, Queensland, Australia; USDA-ARS, United States of America

## Abstract

Originally from Asia, *Rubus niveus* has become one of the most widespread invasive plant species in the Galapagos Islands. It has invaded open vegetation, shrubland and forest alike. It forms dense thickets up to 4 m high, appearing to displace native vegetation, and threaten the integrity of several native communities. This study used correlation analysis between a *R. niveus* cover gradient and a number of biotic (vascular plant species richness, cover and vegetation structure) and abiotic (light and soil properties) parameters to help understand possible impacts in one of the last remaining fragments of the Scalesia forest in Santa Cruz Island, Galapagos. Higher cover of *R. niveus* was associated with significantly lower native species richness and cover, and a different forest structure. Results illustrated that 60% *R. niveus* cover could be considered a threshold for these impacts. We suggest that a maximum of 40% *R. niveus* cover could be a suitable management target.

## Introduction

The invasion of alien plant species is widely accepted to alter ecosystem structure and function, community composition, and species interactions [1], [2], [3], [4]. At the ecosystem level, invasive species can change nutrient cycling, hydrology and fire regimes. At the community level, they may affect vegetation structure, trophic links, availability of space, water, nutrients, light, pollinators and soil properties necessary for germination [5], [6], [7]. There are also cases where population decline of a rare species has been attributed to a community level impact by invasive plants [8].

In invasive species biology, correlations of densities of a whole range of non-native taxa have often been used to infer causality of native biodiversity loss (e.g. [9], [10]). However, caution is needed in inferring causation from correlative studies as it can lead to misunderstanding [11]. Two ways to improve the demonstration of causality in correlative studies of plant invasions are to measure change over time, or to develop a sampling strategy that accounts for both spatial heterogeneity at a landscape scale and stochastic events [12], [13]. While demonstrating impact through experimental manipulation is preferable [14], studies of this type are labour intensive. In the few cases where they have been carried out, results indicate that the mechanism for reduction in the richness and relative abundance of native species can be direct (i.e. competition) or as an indirect consequence of habitat disturbance [15], [16]. However, where no impact is found, it is possible that the long time lag between cause and effect means that evidence may only become apparent in the future [17], [18].

**Figure 1:**
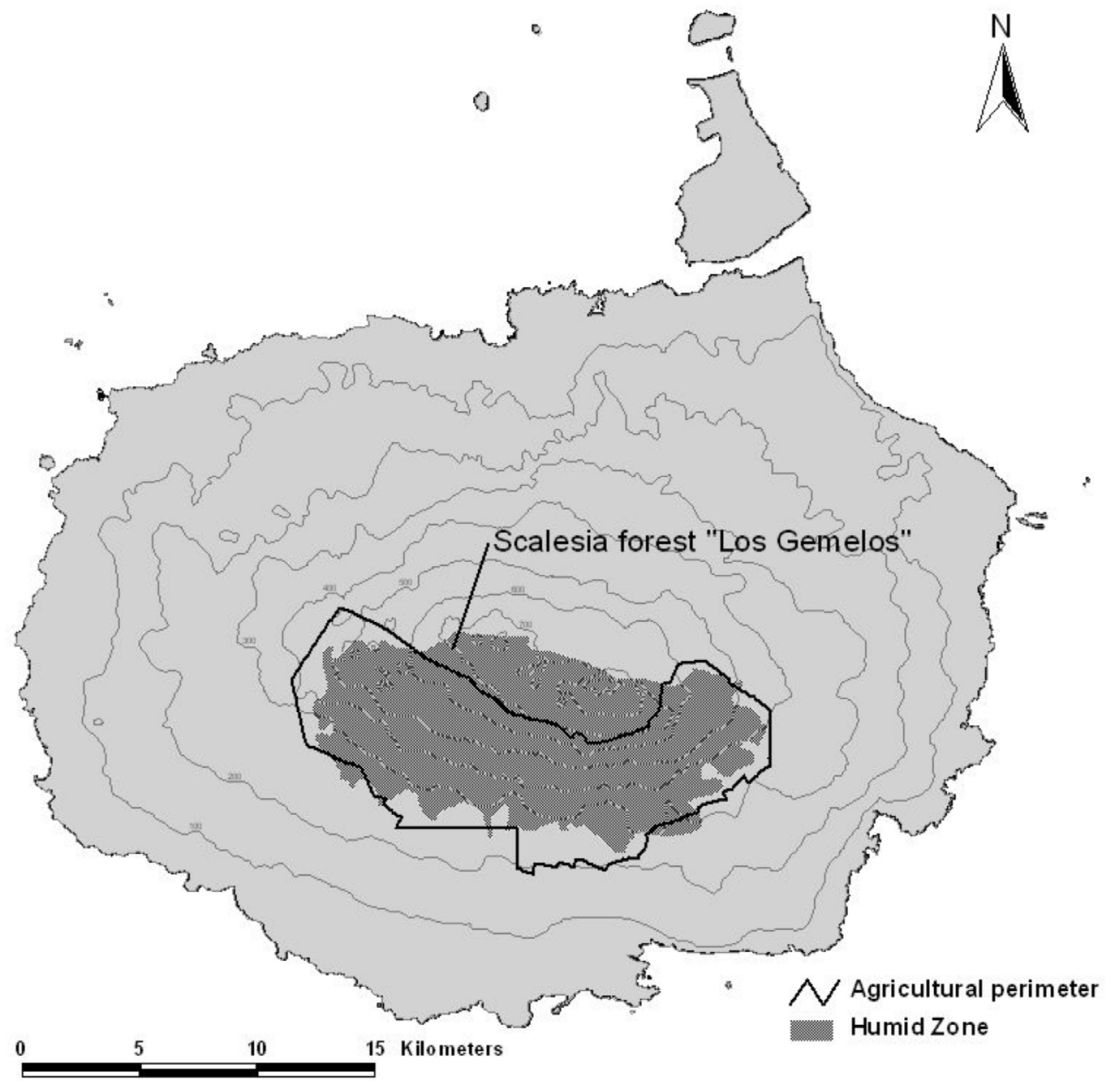
Map of Santa Cruz Island in Galapagos, showing the limit of the humid zone, the mostly overlapping agricultural zone and the location of the Los Gemelos study site.

While the study of invasive species impacts is challenging, it can lead to an increased understanding of the invasion process. This can help improve the effectiveness of invasive species management with the aim of restoring native communities [19], [20], [21], [22], [23], [24]. Useful information includes a better understanding of the effect alien plants have on native communities and ecosystem processes, the threshold densities at which these impacts occur, and the reversibility of these thresholds. An understanding of thresholds allows managers to make realistic decisions on restoration endpoints [25]. Another useful product of impact studies is identification of species or species groups that are most sensitive to impacts. These can be used as indicators by land managers to show that the system is approaching a threshold.

Seventy nine species of *Rubus* are known to be a problem in at least one country in the world [26], [27], [28]. There is anecdotal and quantitative evidence that these species have negative long term impacts on natural ecosystems, preventing the regeneration of native species [29], [30], [31], due to high competitive abilities for resources (such as water, nutrients, space and light), high growth rate, rapid maturity and multiple modes of reproduction [26], [28]. For example, the dense canopy produced by *R. fruticosus* excludes light from the soil surface, effectively dominating other species in the ground stratum [27]. In the early stages of invasion *Rubus* spp. will grow over, or occupy gaps within native vegetation and in later stages they can severely restrict regeneration in native forests [26], [27], [32], [33].

*Rubus niveus* is considered the worst alien plant species in the Galapagos archipelago [34], [35], [36]. In spite of this, to date, no quantitative study has been carried out on its impact there. This species was introduced for agricultural purposes to Santa Cruz Island in the late 1960s and to San Cristóbal Island in the early 1970s [37]. Subsequently, it has been discovered in Floreana Island (2000), two volcanoes of Isabela Island (Sierra Negra and Cerro Azul (2000)), and Santiago Island (2001). *R. niveus* should be considered a transformer species: one that changes the character, condition, form or nature of ecosystems over a substantial area [38]. It can invade grass, bracken, shrub land and forest alike. It forms dense thickets up to 4 m high, displacing native vegetation and threatening native communities such as the *Scalesia pedunculata* forest [39], [40], [41]. In the agricultural zone, *R. niveus* has spread aggressively and as a result the land is useless for agriculture, causing serious economic problems for the farmers. It is already a widespread and serious problem on Santa Cruz and San Cristóbal islands. On the islands where it has been more recently introduced, it is spreading rapidly, especially following the eradication of introduced herbivores, and has proven difficult to eradicate, and expensive to manage [34], [36]. For example, managing *R. niveus* in the remnant *S. pedunculata* forest in Santa Cruz Island, costs the Galapagos National Park Service approximately $US 400 ha^−1^ year^−1^ [42]. However, this investment is made with little understanding of either the impact of *R. niveus* on native plant communities or whether the control is effective in reducing *R. niveus* density to below a threshold of impact on the forest.

**Figure 2:**
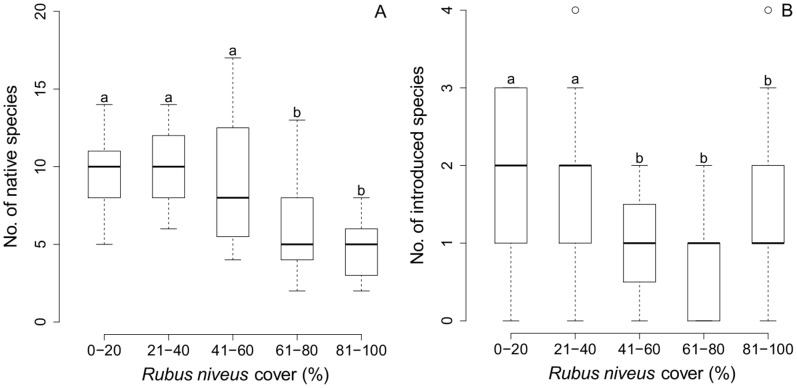
Relationship between *R. niveus* cover and species richness. Lines within the box represent the median values of the number of species found within each cover category; the bottom and top edges of the box represent 25th and 75th percentiles of all data, respectively; the bottom and top bars represent 5th and 95th percentiles. ANOVA-GLM (quasi-Poisson error distribution); native species: F4,119 = 20.83, P<0.001; introduced species: F4,119 = 4.04, P<0.001 (^a,b^denote group differences detected by statistical analyses amongst *R. niveus* cover categories).

Hence, the broad aim of this study was to increase knowledge on the effects of *R. niveus* on the remnant *S. pedunculata* forest at Los Gemelos in Santa Cruz to help improve the effectiveness and efficiency of its management. Our specific objectives were twofold: Firstly, we assessed differences in the vascular plant diversity, cover and structure of native communities of the Scalesia forest at Los Gemelos along a *R. niveus* cover gradient. Secondly, assuming that *R. niveus* actually is the cause of these differences, we determined a potential threshold level of cover for impacts on native plant diversity and cover. We hope that this information can help increase cost effectiveness in conservation management.

For logistical reasons, we were unable to carry out manipulative experiments or repeat measures over time. Instead we did a correlative study, comparing plant species richness, plant species cover, vegetation structure, light availability, pH and soil nutrients with different *R. niveus* densities over the whole extent of the native forest remnant. We adopt a philosophical point of view, that the absence of correlation implies that causation is unlikely [43]. We supplement our results with historic literature to better explain the observed differences in native plant communities.

**Figure 3:**
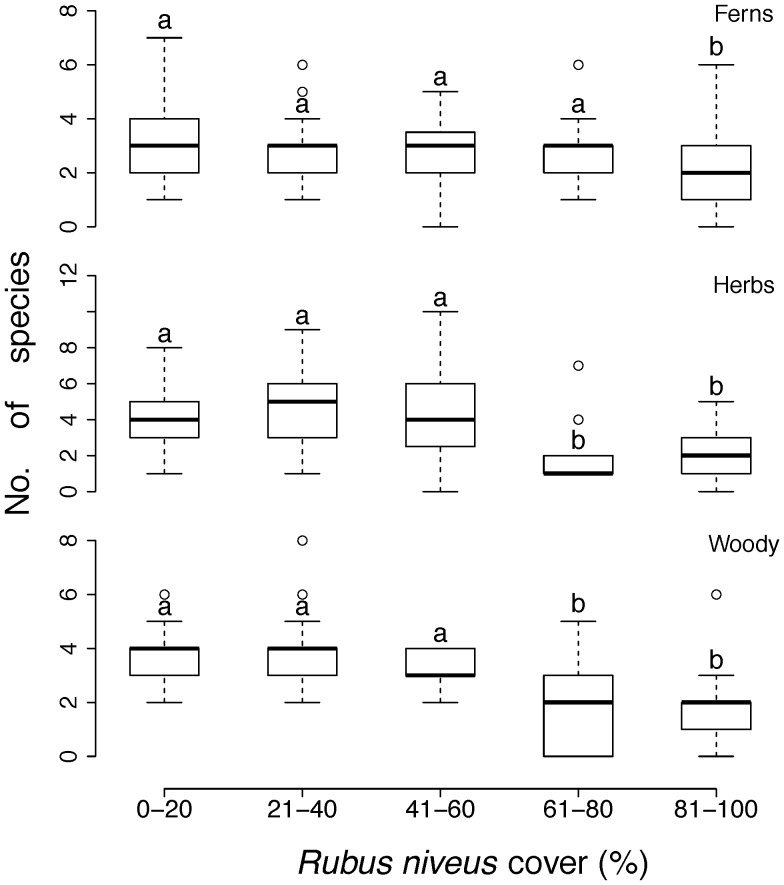
Relationship between *R. niveus* cover categories and total species richness by growth form (vines are included within herbs and woody species include shrubs and trees) and woody (shrubs and trees). Lines within the box represent the median values of the number of species found within each invasion category; the bottom and top edges of the box represent 25th and 75th percentiles of all data, respectively; the bottom and top bars represent 5th and 95th percentiles. ANOVA-GLM (quasi-Poisson error distribution); ferns: F_4,119_ = 3.55, P = 0.009; herbs: F_4,119_ = 11.44, P = 0.009, woody species: F_4,119_ = 13.13, P<0.001 (^a,b^denote group differences detected by statistical analyses amongst *R. niveus* cover categories).

## Materials and Methods

### Study Area on Santa Cruz Island

On Santa Cruz Island, the Scalesia forest is situated within the humid zone (Figure 1), and receives a mean annual precipitation of approximately 1845 mm [44]. Soils are up to 1 m deep, of basaltic origin, well weathered, and sandy loam in texture [45]. This humid zone habitat is the most fertile in the archipelago; the forest is dominated by the endemic tree *Scalesia pedunculata* and constitutes the habitat of many endemic and native species. Historically, Scalesia forest has been cleared extensively so that only 1% of this habitat type remains on Santa Cruz Island [46]. These remnants are invaded by a number of introduced plant species, including *R. niveus*. The Scalesia forest at Los Gemelos, a 200 ha fragment within the Galapagos National Park on Santa Cruz Island, is one of the best remnants of this moist vegetation type in Galapagos [46], [47] (Figure 1).

### Sampling Biological Parameters

A total of 124 plots (2×2 m) were chosen throughout the Los Gemelos study site (ca 200 ha) to represent a variety of cover densities of *R. niveus*. In each plot the composition and structure of the vegetation was assessed using three equally spaced and parallel monitoring transects located 0.5 m apart. Points were taken at 20 cm intervals along these transects, resulting in a total of 30 points per plot. Vegetation height and species cover were assessed with the point-intercept sampling method using a metal rod (1 cm diameter and 3 m high). The rod was marked to distinguish five height classes: 0–0.5, 0.5–1, 1–1.5, 1.5–2 and 2+ m (which included all vegetation up to maximum canopy height). Plant species and their maximum height class intercept at each point were recorded along each transect. To determine plant species richness, the entire plot was searched for species that were not recorded in the transect monitoring.

**Figure 4:**
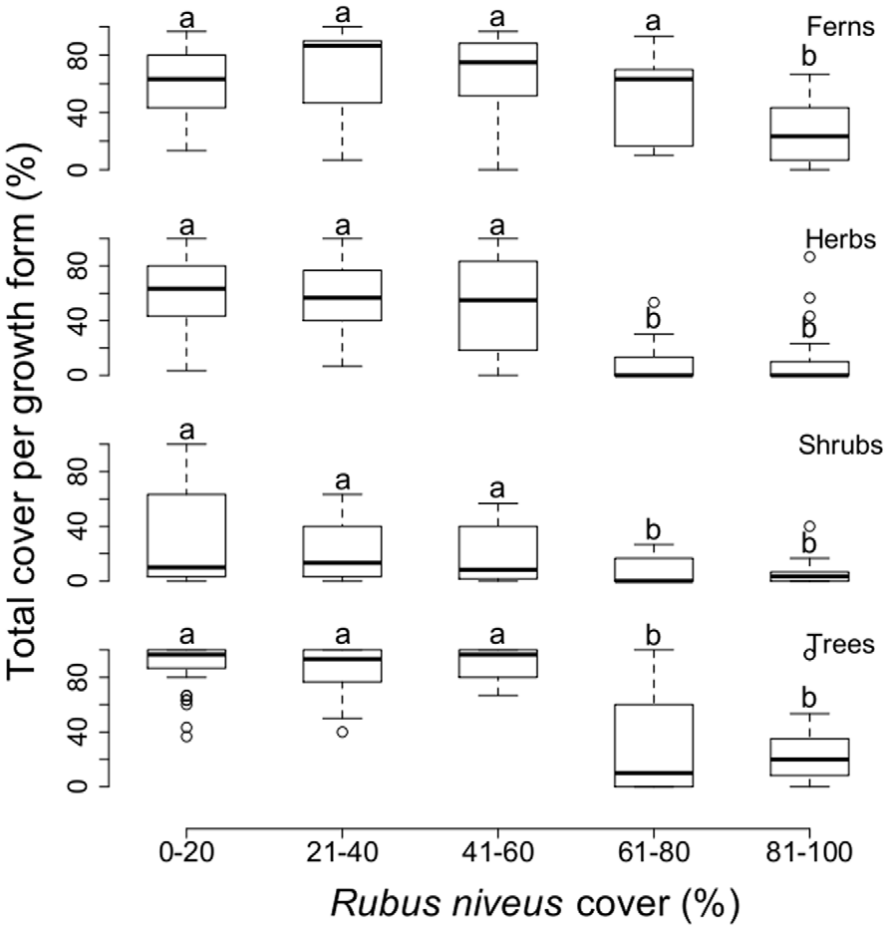
Relationship between *R. niveus* cover categories and total species cover by growth form. Lines within the box represent the median values of cover within each invasion category; the bottom and top edges of the box represent 25th and 75th percentiles of all data, respectively; the bottom and top bars represent 5th and 95th percentiles. ANOVA-GLM (quasi-Binomial error distribution); ferns: F_4,119_ = 9.54, P<0.001; herbs: F_4,119_ = 20.86, P<0.001, shrubs: F_4,119_ = 7.27, P<0.001; trees: F_4,119_ = 35.41, P<0.001 (^a,b^denote group differences detected by statistical analyses amongst *R. niveus* cover categories).

### Sampling Abiotic Parameters

Light intensity was measured using a digital light meter at 0.5 and 2 m height (5 readings in each plot). Additionally, soil samples were taken with a 10 cm deep, 4 cm diameter soil core at plot centres. Soil samples were taken from sites with high and low *R. niveus* cover (>80% cover, n = 11, <20% cover, n = 11 respectively) and were sent to the University of Azuay, Cuenca, Ecuador for analysis of total Ca, C, K, N total, NH_4_, NO_3_, P and pH.

### Analysis

*Rubus niveus* cover was used as the explanatory variable to investigate the relationship with the biological and abiotic response variables (plant species richness, plant species cover, vegetation structure, light availability, pH and soil nutrients). For these analyses, *R. niveus* cover was grouped into five continuous categories (low: 0–20%, n = 41; medium: 20–40%, n = 29; 40–60%, n = 16; high: 60–80%, n = 9; 80–100%, n = 29).

**Figure 5:**
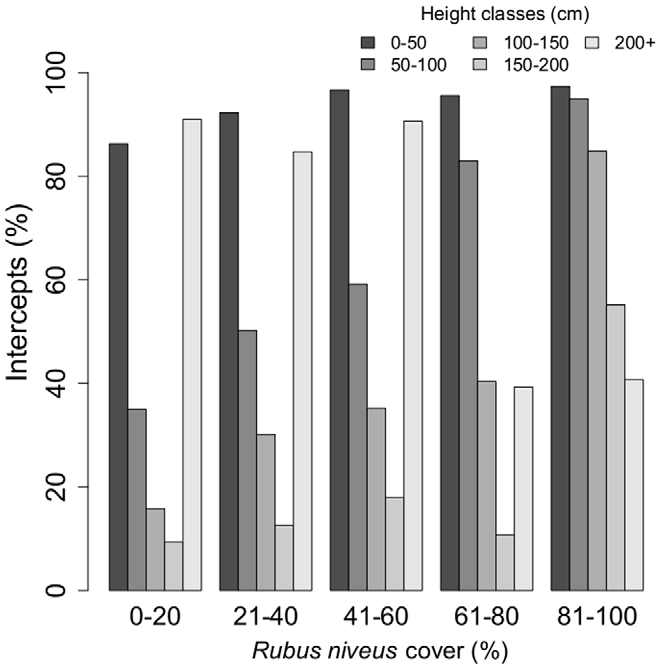
Vertical structure description of the forest using percent of intercepts by height class within each of the *R. niveus* cover categories. Height classes can sum to values that exceed 100% because of the multiple strata in the forest.

Species richness (the number of species present in each plot), species cover (the frequency of species occurrence in each plot), and vegetation structure were calculated for each plot and compared between the five *R. niveus* cover categories.

Generalized Linear Models (GLM) were used to assess the relationship between the *R. niveus* cover categories and the abiotic and biological parameters. ANOVAs were used to test the significance of parameters. SIMPER analysis (in PRIMER) was used to determine the contribution of each species to the average Bray-Curtis dissimilarity index between two *R. niveus* cover categories that represent the grouping of the 5 categories used in previous analyses (0–60%, n = 86; 60–100%, n = 38). This grouping into two was based on previous statistical analysis that indicated a significant difference in species diversity when *R. niveus* cover was 60% or greater. This method of analysis determines which species contribute most to the differences, i.e. which species cover was correlated most closely with *R*. *niveus* [48].

**Figure 6:**
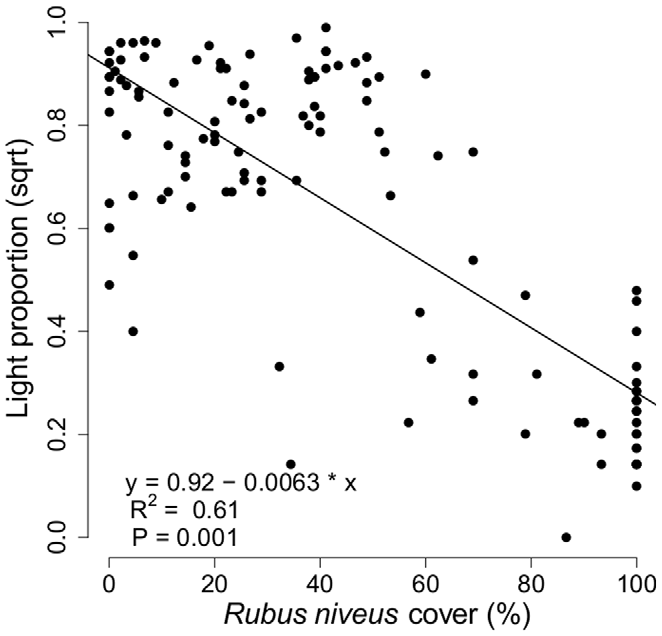
Relationship between *R. niveus* cover categories and the proportion of light reaching the understorey (proportion = light intensity at 0.5 m/light intensity at 2 m).

## Results

### Biological Parameters

#### a) Plant species richness

In total, 56 vascular plant species were recorded across all plots, comprising 47 native and 9 introduced species. Grouped by growth form there were 15 ferns, 23 herbs, four vines, four shrubs and 10 trees. Approximately 37% of species occurred at all stages of *R. niveus* invasion; 10% of species, of which all were native, were only recorded at low *R. niveus* cover category (<20%).

A significant decline in both native and introduced species richness was correlated to increasing *R. niveus* cover (ANOVA-GLM; native species: F_4,119_ = 20.83, P<0.001; introduced species: F_4,119_ = 4.04, P<0.001) (Figure 2A, 2B). Sites with high *R. niveus* cover (>60%) contained on average 56% fewer species than sites with medium to low cover (<60%). Although fewer introduced species were recorded than native species (9 introduced species, 47 native), both groups showed proportionally lower levels of species richness when *R. niveus* cover was above 60%.

In addition, each of the three key growth forms had lower species richness at higher *R. niveus* cover categories although this pattern varied with growth form (Figure 3). The number of fern species was significantly less in sites where *R. niveus* cover was above 80% (ANOVA-GLM, F_4,119_ = 3.55, P = 0.009) whereas herb and woody species richness showed a significant decrease when *R. niveus* cover above 60% (ANOVA-GLM; herbs: F_4,119_ = 11.44, P = 0.009, woody species: F_4,119_ = 13.13, P<0.001) (Figure 3). On average, fern species richness was reduced by 31%, herb species richness by 54% and tree species richness by 48% when comparing the number of species between the lowest and highest *R. niveus* cover categories.

**Table 1:** Results of similarity analysis (cover and presence SIMPER analysis) for vascular plant species.

Species	Growth form	Origin	Average cover (%)			**Contribution
			*0-60	*60-100		(%)
Cover dissimilarity = 85.85%						
*Scalesia pedunculata*	tree	endemic	56.5		13.6	8.6
*Cestrum auriculatum*	tree	introduced	28.6		6.2	6.3
*Asplenium auritum*	fern	native	23.9		5.2	5.8
*Blechnum occidentale*	fern	native	15.5		13.5	5.1
*Ichnanthus nemorosus*	herb	native	18.8		1.5	4.9
*Chiococca alba*	shrub	native	16.5		3	4.5
*Thelypteris pilosula*	fern	native	10.4		4.1	4
*Passiflora colinvauxii*	vine	endemic	6.9		8.6	3.9
*Blechum pyramidatum*	herb	native	11.3		0.6	3.6
*Passiflora edulis*	vine	introduced	8.2		6.5	3.5
			**Presence (%)**			
			* **0-60**	* **60-100**		
Presence/absence dissimilarity = 71.41%						
*Scalesia pedunculata*	tree	endemic	80.2	36.8		4.8
*Cestrum auriculatum*	tree	introduced	65.1	34.2		4.7
*Asplenium auritum*	fern	native	64	34.2		4.6
*Ichnanthus nemorosus*	herb	native	54.7	18.4		4.3
*Thelypteris pilosula*	fern	native	47.7	44.7		4.2
*Chiococca alba*	shrub	native	48.8	31.6		4.1
*Blechnum occidentale*	fern	native	38.4	42.1		4
*Psychotria rufipes*	shrub	native	45.3	15.8		3.9
*Passiflora colinvauxii*	vine	endemic	60.5	76.3		3.9
*Doryopteris pedata*	fern	native	44.2	18.4		3.8
*Passiflora edulis*	vine	introduced	32.6	36.8		3.7
*Tournefortia rufo-sericea*	shrub	native	36	26.3		3.5

Species are listed in descending order according to contribution to compositional dissimilarities between two broad *R. niveus* cover categories (0–60%, n = 86; 60–100%; n = 38). Only species contributing up to approximately 50% of the total are shown.

* *R. niveus* cover categories.

** Individual species contribution to dissimilarities between cover categories.

#### b) Plant species cover

Species cover of all of the growth form groups showed a significant difference with *R. niveus* cover (Figure 4). Fern cover was significantly less in sites where *R. niveus* cover was above 80% (ANOVA-GLM; F_4,119_ = 9.54, P<0.001). On average, fern cover was about 48% less in sites with high cover. Herb, shrub and tree cover were also significantly lower when *R. niveus* cover was over 60% (ANOVA-GLM; herbs: F_4,119_ = 20.86, P<0.001, shrubs: F_4,119_ = 7.27, P<0.001; trees: F_4,119_ = 35.41, P<0.001). On average, cover values of herbs, shrubs and trees were 82, 78 and 68% less respectively with >60% cover of *R. niveus*. The canopy layer (>2 m) was also much less.

According to the similarity analysis (SIMPER analysis results) in Table 1, site dissimilarity was better explained by species cover (85.8%) than species presence/absence (71.4%). A lower average cover of most species was associated with high *R. niveus* cover (>60%) when compared to sites with medium to low covers (<60%). The endemic *Scalesia pedunculata* and the introduced *Cestrum auriculatum* trees exhibited the highest cover in the study area. These two species together contributed 14.9% of the total percentage of dissimilarity in species cover. The most notable difference in cover between sites with low and high cover of *R. niveus* was seen in the native herbs *Ichnanthus nemorosus* and *Blechum pyramidatum,* and the native shrub *Chiococca alba* (92%, 95% and 82% lower respectively). Conversely, a high cover of the endemic vine *Passiflora colinvauxii* was associated with high *R. niveus* cover.

#### c) Vegetation structure

A slight change in vegetation structure was associated with increased *R. niveus* cover (Figure 5). Vegetation in areas with a *R. niveus* cover less than 20% was dominated by an under-storey (0–0.5 m) composed mainly of ferns (*Asplenium auritum* and *Blechnum occidentale*) and herbs (*Ichnanthus nemorosus* and *Blechum pyramidatum*) and a prominent canopy (>2 m) dominated by the endemic tree *Scalesia pedunculata*. Mid-storey vegetation (0.5–2 m) was almost absent and dominated by a few shrubs such as *Chiococca alba* and *Tournefortia rufo–sericea*). In contrast, with *R. niveus* cover above 60%, the under-storey and the mid-storey layers were dominated by *R. niveus*. The density of the canopy layer decreased from 80% to 40%.

### Abiotic Parameters

A linear model showed a negative relationship between *R. niveus* cover and the proportion of light intensity reaching the understorey (Figure 6). At high *R*. *niveus* cover (>80%), light intensity reaching the under-storey was about 94% lower than the amount of light reaching the mid-storey. In contrast, in sites with a low cover of *R. niveus* (<20% cover), the light intensity reaching the under-storey was about 45% of that reaching the mid-storey.

In general there were no significant differences in soil nutrient composition or pH between sites with high (>80%) and low (<20% cover) *R. niveus* cover (Table 2). Although the mean values of soil parameters in sites with high *R. niveus* cover were higher than those with low cover, the difference was significant only when comparing NO_3_ (t-test: df = 10, t = 2.37, p = 0.039) and pH (t-test: df = 10, t = 2.37, p = 0.001).

## Discussion

### Biotic and Abiotic Differences Across a *R. niveus* Cover Gradient

Despite the great threat posed by invasive plants to the natural ecosystems in Galapagos, almost no impact studies have been carried out there to date [49], [50], [51]. This study focussed on one of the worst weeds in Galapagos, and its impact on one of the most threatened natural vegetation types, with an aim to determine if the information can help in improving the effectiveness of habitat restoration. The invasion of *R. niveus* in the Scalesia forest began no longer than 15 years ago [52]. Therefore, the documented high densities of *R. niveus* have developed recently over much of the study area [35], [37] so it is not known if the patterns we found may change with time. We also acknowledge that our study covered a limited area, a single sampling period at that it was correlatory in nature.

We found that a high cover of *R. niveus* was associated with lower plant species richness of both native and non-native species, lower cover, and a simplified vegetation structure. While we acknowledge that the data are correlatory, they support perceptions that *R. niveus* invasion is harmful to the Galapagos vegetation communities [35] and concur with current understanding of the impacts of invasive plants on natural ecosystems and in particular to those associated with invasion by the genus *Rubus* worldwide.

The strong correlation presented here demonstrates a probable negative impact of *R. niveus* cover on the species richness of the resident plant community of the Scalesia forest. Species richness in each growth form was significantly lower with higher *R. niveus* cover, and overall more than halved when *R. niveus* cover was above 60%. This indicates that the threat of *R. niveus* may be generalized across all life forms in the recipient community, although ferns may slightly more resilient.

Native species composition (measured by cover and similarity) appears to be slightly more sensitive to higher *R. niveus* cover than richness. Tree species contributed most to compositional dissimilarities between sites with high *R. niveus* cover and sites with medium to low cover, especially the endemic *Scalesia pedunculata,* and the natives *Chiococca alba*, *Ichnanthus nemorosus* and *Blechum pyramidatum*. The anomaly is the endemic vine *Passiflora colinvauxii* whose presence and cover was correlated positively with *R. niveus* cover. *Passiflora colinvauxii* is present in even the most disturbed systems across Santa Cruz (M. Trueman personal communications).

In terms of abiotic parameters, sites highly infested by *R. niveus* had a slightly higher amount of nutrients available in the soil. Changes in soil properties are often recorded in invaded systems. This may be due to an altered species diversity or composition [53], [54], [55], or due to the invasive species itself, often noted with the invasion of nitrogen fixing species that helps in the invasion process at the detriment of the native flora [53], [54], [55]. Higher nutrient concentrations in the topsoil have also been reported in areas infested by some invasive *Rubus* spp. [26], [56], [57], thus it might have been expected to find a more profound difference in soil nutrients between sites with low and high cover of *R. niveus*. It is possible that the invasion of the Scalesia forest has been too recent [52] to have a major effect on soil nutrient status, or there may be one or more over-riding factors that mask any correlatory patterns.

### Ecological Implications for the Scalesia Forest

Species diverse communities are often thought to be highly competitive and resistant to invasion [58], [59], [60], [61]. In Galapagos the flora is depauperate in shrub and tree species [53], [54] and forests such as the one studied here have a very simple vegetation structure and species composition [51]. The Scalesia forest is dominated by the short lived tree *Scalesia pedunculata* and a few sparse shrub species, suggesting that there are vacant niches [52]. In addition, *S. pedunculata* experiences a periodic massive dieback as a mechanism for regeneration [49], [50]. As invasive plants normally fill unoccupied canopy spaces and may spread rapidly with forest disturbance such as treefall events, storm damage and stand dieback [62], it would suggest that this forest type is highly susceptible to invasion.

**Table 2:** Summary of the soil physical characteristics between sites in lowest (<20%, n = 11) and highest (>80%, n = 11) *R. niveus* categories.

Physical parameter	Mean value (ppm)		df	t-value	p-value
	>80%	<20%			
Ca	1147.9	1019.3	10	1.03	0.325
C	170.3	154.3	10	0.87	0.402
K	1.1	1.8	10	−0.7	0.498
N total	966.1	649.3	10	1.15	0.275
NH_4_	247.6	152.4	10	0.75	0.466
NO_3_	4	1.8	10	2.37	0.039*
P	28.3	22.2	10	0.86	0.407
pH	6.5	6.9	10	−4.35	0.001*

*Denotes significance of differences between mean values.

We believe that *R. niveus* may not be directly responsible for the differences in biological parameters reported here, but it probably contributes to a negative feedback cycle. *Rubus niveus* is a gap loving species, and although the canopy cover of an intact Scalesia forest is almost 100%, it is not dense and there is sufficient light to allow the existence of understorey growth forms [51], [63]. Dense stands of *R. niveus* are often found in open canopy areas and while there is no evidence that *R. niveus* is causing the mortality of mature canopy species in the Scalesia forest, it is preventing species recruitment. The invasion process may also be helped by the dieback events mentioned above.

While *R. niveus* has only reached high densities in the Scalesia forest in the last 5 years [35] we can already see that these high densities are correlated to low light levels in the ground stratum, causing changes in the micro-climate normally present in the forest. An abundance of canes and a dense foliar layer produced by *R. niveus* creates a dark and wet habitat that is unlikely to be suitable for the recruitment of shade intolerant native species [64], which dominate this vegetation type and include *Scalesia pedunculata* [51], [65], [66]. In addition, *R. niveus* is a scrambling species that may smother native plants, leading to a dense monotypic thicket with little other vegetation present. The ability to regenerate also depends on the available seed bank. It logically follows that as *R. niveus* density increases, the cover of native plants will decrease and so will their seed production. In addition to effects on individual species, a reduction in species richness and cover may also change ecosystem functioning (e.g. mutualisms such as pollination and seed dispersal) and services [4], [67]. A similar pattern has been observed in another invaded upland community on the island, where a 7-year study showed that the presence of *Cinchona pubescens* was correlated with a reduction in species diversity and cover of most species by at least 50% within the invaded area, compared to the control site [62].

### Management Implications

A consistent result from most of the statistical analyses presented here is that significant differences in species richness cover and vegetation structure are associated with a high cover of *R. niveus* (>60%). This could be considered a “threshold value” for impact, thus providing a guideline for management of the species if causation can be proven through an experimental approach. Even though the current study relies on correlatory evidence, we suggest that it provides a useful starting point for rethinking the management approach to the *R. niveus* invasion in Los Gemelos. In addition, this threshold is based only on work on vegetation and studies are needed to determine if the same patterns exist for other groups such as invertebrates and birds.

If the aim of management is to reduce *R. niveus* below a threshold of impact on the ecology of the system, we suggest that a conservative maximum of 40% *R. niveus* cover could be a suitable management target. This management target could also act as a guide for the development of any biological control agent. In the case that land managers in the Galapagos have difficulty estimating 40% cover, our results indicate that three species could potentially act as indicators of ecosystem-level impact. The absence of *Chiococca alba*, *Ichnanthus nemorosus* and *Blechum pyramidatum*, may indicate the threshold has been passed and hence provide a useful rule of thumb. The shrub, *Chiococca alba* may be the best choice for an indicator because it is long-lived and can be easily identified by people working in land management.

This management aim contrasts sharply with the current management objective which is to eliminate *R. niveus* from the area. The intensive management approach is partly based on the fact that the area is a tourist site and the forest along the trails is kept ‘clean’ in order to demonstrate the original highland ecosystem to visitors. It is also based on the fact that there have been many successful mammalian eradication programmes in Galapagos (e.g. [68], [69]) leading to the false expectation that all invasive species can be eradicated. However, the expense of this approach ($US 400 ha^−1^ year^−1^) means that only a small area can be under active management, and the resultant disturbance of this highly intensive approach facilitates further invasion of *R. niveus* and other species.
